# Novel Tunable Pseudoresistor-Based Chopper-Stabilized Capacitively Coupled Amplifier and Its Machine Learning-Based Application

**DOI:** 10.3390/mi16091000

**Published:** 2025-08-29

**Authors:** Mohammad Aleem Farshori, M. Nizamuddin, Renuka Chowdary Bheemana, Krishna Prakash, Shonak Bansal, Mohammad Zulqarnain, Vipin Sharma, S. Sudhakar Babu, Kanwarpreet Kaur

**Affiliations:** 1 Department of Electronics and Communication Engineering, Jamia Millia Islamia, New Delhi 110025, India; mhdaleemfarshori218@gmail.com (M.A.F.); mnizamuddin1@jmi.ac.in (M.N.); 2 Department of ECE, NRI Institute of Technology, Vijayawada 521212, India; renukachowdarybheemana@gmail.com; 3 Department of Electronics and Communication Engineering, Chandigarh University, Gharaun, Mohali 140413, India; 4 Integrated Circuits Group, Eindhoven University of Technology, 5612 AZ Eindhoven, The Netherlands; zulqar11@gmail.com; 5 Department of Electronics and Communication Engineering, Raj Kumar Goel Institute of Technology, Ghaziabad 201017, India; vksharma@zhcet.ac.in; 6 Vignan’s Institute of Information Technology, Duvvada, Visakhapatnam 530049, India; sudha.227@gmail.com; 7 Department of Electronics and Communication Engineering, Thapar Institute of Engineering and Technology, Patiala 147004, India

**Keywords:** CMRR, bio-signal, chopper stabilization, pseudoresistor, FinFET, machine learning

## Abstract

This work presents a high-common-mode-rejection-ratio (CMRR) and high-gain FinFET-based bio-potential amplifier with a novel CMRR reduction technique. In this paper, a feedback buffer is used alongside a capacitively coupled chopper-stabilized circuit to reduce the common-mode signal gain, thus boosting the overall CMRR of the circuit. The conventional pseudoresistor in the feedback circuit is replaced with a tunable parallel-cell configuration of pseudoresistors to achieve high linearity. A chopper spike filter is used to mitigate spikes generated by switching activity. The mid-band gain of the chopper-stabilized amplifier is 42.6 dB, with a bandwidth in the range of 6.96 Hz to 621 Hz. The noise efficiency factor (NEF) of the chopper-stabilized amplifier is 6.1, and its power dissipation is 0.92 µW. The linearity of the parallel pseudoresistor cell is tested for different tuning voltages (V_tune_) and various numbers of parallel pseudoresistor cells. The simulation results also demonstrate the pseudoresistor cell performance for different process corners and temperature changes. The low cut-off frequency is adjusted by varying the parameters of the parallel pseudoresistor cell. The CMRR of the chopper-stabilized amplifier, with and without the feedback buffer, is 106.9 dB and 100.3 dB, respectively. The feedback buffer also reduces the low cut-off frequency, demonstrating its multi-utility. The proposed circuit is compatible with bio-signal acquisition and processing. Additionally, a machine learning-based arrhythmia diagnosis model is presented using a convolutional neural network (CNN) + Long Short-Term Memory (LSTM) algorithm. For arrhythmia diagnosis using the CNN+LSTM algorithm, an accuracy of 99.12% and a mean square error (MSE) of 0.0273 were achieved.

## 1. Introduction

Health monitoring devices, such as electrocardiograms (ECGs), electroencephalograms (EEGs), and electromyograms (EMGs), have recently garnered research interest in the effort to prevent health complications and reduce mortality rates. As integrated circuit (IC) processes evolve, there is a growing demand for high-gain and low-power bio-sensing devices. Also, noise has a considerable impact on biomedical signal processing, particularly flicker noise, as its amplitude is comparable to that of the biomedical signals [1]. Chopper-stabilized amplifiers reduce the effect of low-frequency flicker noise and DC offset while simultaneously boosting the CMRR of the circuit. Since the CMRR depends on both resistance and capacitance values, both must be accurately tuned to provide a high CMRR. Fully differential difference amplifiers (FDDAs) are used instead of three-amplifier topologies to boost the CMRR, applying parasitic capacitance reuse techniques to optimize noise [2]. Multichannel neural amplifiers, which use common-mode feedback through preamplifier supply rails, improve both the common-mode range and CMRR [3]. Cascading three amplifier stages to create a logarithmic programmable gain amplifier (LPGA) is another technique to enhance the CMRR while reducing the common-mode gain [4]. A chopper-stabilized amplifier with a programmable bandwidth, using Miller compensation techniques, is discussed in [5]. The low cut-off frequency of chopper-stabilized amplifiers was reduced in [6] using cross-coupled positive feedback circuitry. Low-power, high-CMRR neural amplifiers are designed using a bandwidth-boosted folded cascade OTA (operational transconductance amplifier) [7]. Biomedical signals such as ECG, EEG, and EMG vary in amplitude (typically in the millivolt to microvolt range) and frequency (0–500 Hz) [8]. The low amplitude of biomedical signals makes them vulnerable to noise, necessitating additional circuitry to eliminate noise. For proper design and optimization of biomedical circuits, a trade-off between power, gain, and noise is necessary, and the g_m_/I_d_ biasing technique is preferable [9]. Flicker noise, or 1/f noise, is most prominent at low frequencies [1], where most biomedical signals exist. Flicker noise can be reduced by employing chopper-stabilized amplifiers [10]. The noise efficiency factor (NEF) is used to measure noise performance in biomedical circuits, and a low NEF value is desired for better noise handling. In addition, power is also one of the most important amplifier performance parameters and can be optimized through various techniques [11]. Nanodevices can replace outdated CMOS technology for better short-circuit current handling and less power leakage. FinFET is a novel device that offers better control over the channel, resulting in low leakage and a high switching speed. FinFET has fewer short-channel effects, especially DIBL [12], which leads to low power dissipation. FinFET has been used in various analog and digital circuits, such as SRAM [13] and Schmitt triggers [14], consistently outperforming planar MOS technology. For IC design, area optimization is of great concern. Recently, conventional resistors have been replaced by MOS-based pseudoresistors, which require less area [15]. When a MOS transistor is biased in the sub-threshold or weak inversion region, it can function as a resistor, commonly referred to as a pseudoresistor. The pseudoresistor covers less area compared to traditional resistors, making it an attractive option for IC design [15]. MOS-based pseudoresistors can be tuned by applying a bias voltage to the gate terminal, providing greater control over the resistance value [16]. Most filters require a high RC time constant, which can be implemented using a pseudoresistor, all while consuming relatively less area. A unique property of the pseudoresistor is its ability to be tuned for bandwidth by applying a voltage to the gate terminal. One drawback of the pseudoresistor is that it can be difficult to achieve high resistance values when using advanced technology. Pseudoresistor implementations often suffer from the variable V_gs_ problem, which causes a change in resistance value, leading to gain-related distortion. To eliminate this issue, a circuit of series-connected pseudoresistors with a source follower was designed [17].

In this work, a self-biased inverter-based amplifier is used as the core amplifier for analog front-end design. The self-biased inverter amplifier consumes less area and also provides high gain. Traditional passive resistors are bulky and require a large area, making them unsuitable for integrated circuit design. Also, a parallel cell configuration of pseudoresistors is used as a replacement for passive resistors. By varying the tunable voltage (V_tune_) of the pseudoresistor, it is demonstrated that the low cut-off frequency of the chopper-stabilized amplifier can be adjusted. Additionally, the low cut-off frequency can be varied by altering the number of pseudoresistor cells. A feedback buffer is also employed alongside the pseudoresistor cell to reduce the common-mode gain and enhance the CMRR. The feedback buffer serves a dual purpose, increasing the CMRR while also reducing the low cut-off frequency, as shown mathematically in this work. Furthermore, to capitalize on the advantages of FinFET technology over conventional CMOS technology, all the circuit components in our work are designed using novel FinFET-based technology. This paper is divided into six sections. Section 1 provides an introduction to the work. Section 2 gives an overview of the material and methods. Section 3 presents the results. Section 4 explores the machine learning-based application of biomedical signals. Section 5 discusses the proposed work in comparison with existing work. Finally, Section 6 concludes the paper.

## 2. Materials and Methods

### 2.1. Capacitively Coupled Chopper-Based Amplifier

**Figure 1:**
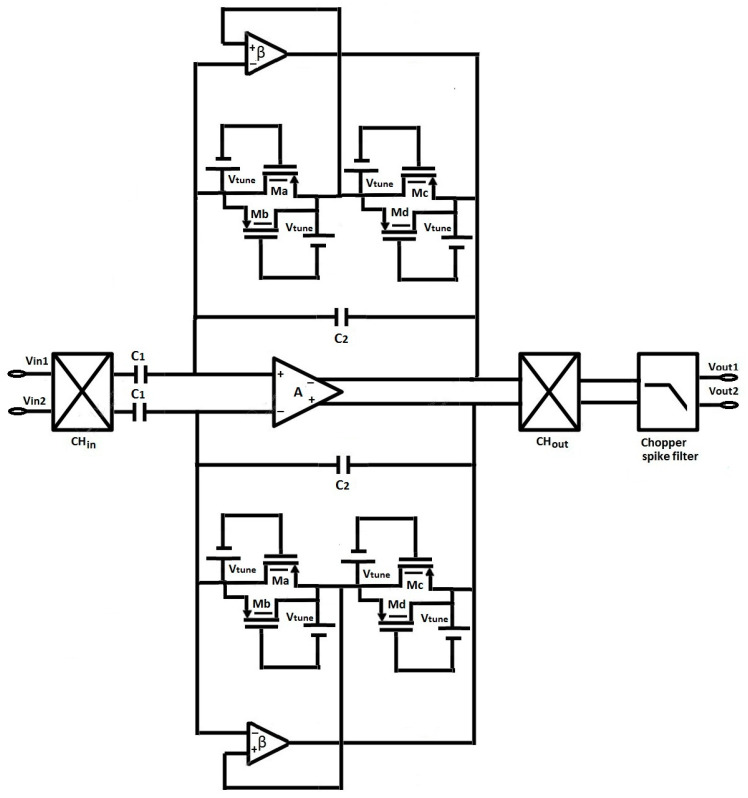
Schematic of the proposed capacitively coupled chopper-stabilized amplifier.

**Figure 2:**
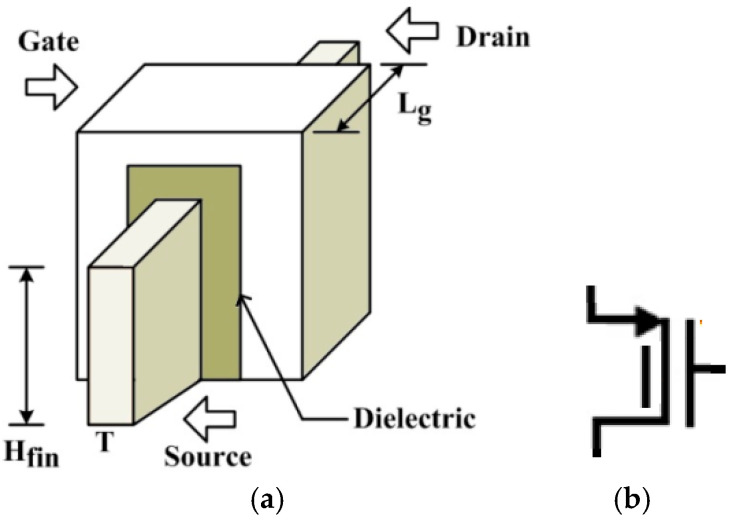
(**a**) Geometry of FinFET. (**b**) Symbol of pFinFET.

In this section, a capacitively coupled chopper amplifier, as shown in Figure 1, is designed using a parallel tunable pseudoresistor cell, which is discussed later in Section 2.2. The parallel tunable pseudoresistor cell replaces the high-value resistor of the capacitively coupled chopper amplifier in the feedback loop, reducing the chip area and simplifying the fabrication process. The parallel tunable pseudoresistor cell is designed using FinFET, and its resistance value is sufficiently high to create a low-frequency pole in the biomedical signal range. FinFET’s 3D structure, with its vertical channel, carries a high current density, which can be modulated by using a large number of fins. The geometry of the FinFET device is shown in Figure 2a, and its symbol is shown in Figure 2b. The effective width of FinFET is given by the equation:

W_eff_ = 2·H_fin_ + T (1)

where H_fin_ = height of fin; T = thickness of fin. FinFET has both vertical and horizontal components of effective width, as shown in Equation (1). Generally, the height of the fin is kept higher than the thickness of the fin to mitigate short-channel effects. However, FinFETs with gate lengths below 10 nm face several challenges, including but not limited to increased electromigration, complex fabrication processes, threshold voltage variation, and other issues.

The capacitively coupled chopper amplifier, as shown in Figure 1, consists of a modulator (CH_in_) that shifts the original signal to a higher frequency, keeping the flicker noise in the low-frequency range. The output chopper demodulator (CH_out_) performs the opposite operation, ultimately keeping flicker noise at bay in the original input signal. As the input capacitor C_1_ is placed just after CH_in_, as shown in Figure 1, it will not affect the CMRR value, but will introduce non-linearity in the circuit. The mid-band gain is controlled by the feedback capacitor ratio C_1_/C_2_, and the values of these capacitors are chosen to maintain a high gain while remaining compatible with IC design. The chopper circuit introduces spikes in the output, which can be eliminated through RC filters designed using FET-based switches and capacitors. In every input clock cycle, there is a variation in voltage across C1 between positive and negative values, which results in the flow of charging and discharging current from the input signals (vin1 and vin2). This charging and discharging of input capacitor C1 in combination with input chopper modulator (CH_in_), as shown in Figure 1, results in the formation of a switch capacitor resistor with an input resistance value expressed as:

(2) $$R_{in}=\;\frac{1}{2\cdot C_{1}\cdot f_{chop}}$$

where $f_{chop}$ is the chopping frequency of CH_in_. This expression can be derived by modeling the charging and discharging of the input capacitor *C*_1_ in each clock cycle. The average charge transferred in each switching cycle is

$$Q=C_{1}(vin1-vin2)$$

and the corresponding average current is

$$I=Q\cdot f_{chop}=C_{1}\cdot f_{chop}.(vin1-vin2)$$

Applying Ohm’s law, the equivalent input resistance is therefore

$$R_{in}=\frac{\left(vin1-vin2\right)}{I}=\frac{\left(vin1-vin2\right)}{C_{1}\cdot f_{chop}\cdot (vin1-vin2)}$$

This simplifies to Equation (2), with the factor of 2 arising from the two non-overlapping phases of the chopping operation.

A high C_1_ is needed for high gain, which in turn requires a large area. Additionally, a large C_1_ reduces the input impedance, potentially causing signal loss. High chopping frequencies significantly affect the input impedance value, necessitating an impedance-boosting loop with positive feedback, though this increases the input-referred noise of the amplifier. The input-referred noise of a capacitively coupled amplifier is given by:

(3) $$v_{ni\;}^{2}=\;\left(\frac{C_{1}+C_{2\;}+{\;C}_{in}}{C_{1}}\right).\bar{v_{ni,A}^{2}}$$

where C_1_ and C_2_ are the input terminal capacitance and feedback capacitance, respectively, C_in_ is the total input capacitance of the inverter-based amplifier (A) and feedback buffer (β), and $\bar{v_{ni,A}^{2}}$ is the input-referred noise of the inverter-based amplifier A, as shown in Figure 1. Noise and gain are crucial parameters in determining the values of capacitors C_1_ and C_2_. NEF refers to the ability of the circuit to handle noise, and it is one of the important figures of merit in bio-potential amplifiers. NEF is given by the equation:

(4) $$NEF=V_{i,rms}\sqrt{\frac{{2I}_{t}}{4\pi \cdot V_{T}\cdot k\cdot T\cdot BW}}$$

where $V_{i,rms}$ is the total input referred noise, $V_{T}$ is the thermal voltage, k is Boltzmann constant, BW is bandwidth, and $I_{t}$ is the total current drawn from the voltage source.

### 2.2. Pseudoresistor

**Figure 3:**
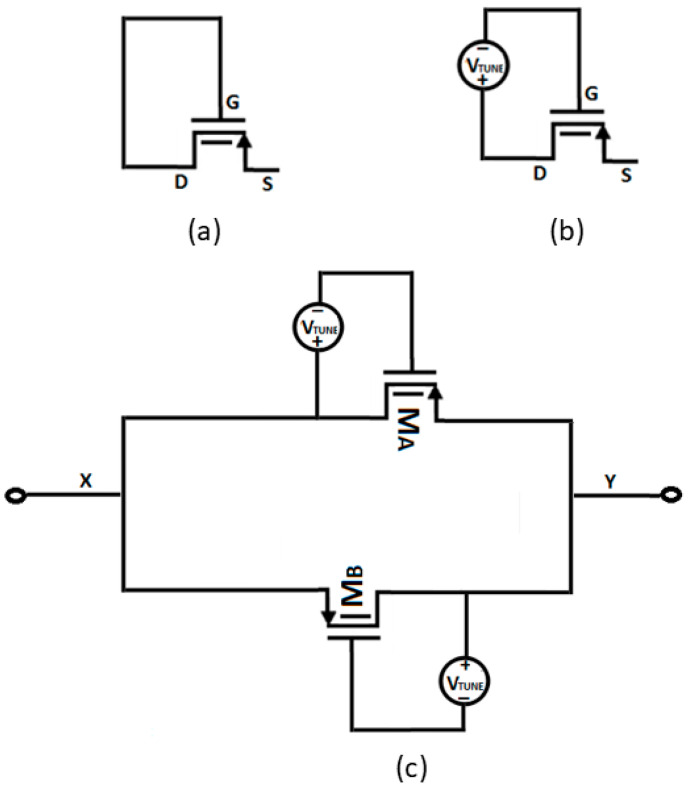
Schematic of (**a**) a pFinFET-based pseudoresistor. (**b**) Tunable configuration of a pFinFET-based pseudoresistor. (**c**) Parallel cell configuration of a tunable pseudoresistor.

Pseudoresistors can be voltage- or current-controlled, with resistance values exceeding 100 GΩ, though this resistance value is not easily controlled. Resistance can be tuned by applying a bias voltage between the gate and source or between the gate and drain, with the value of the bias voltage determining whether the pseudoresistor operates in strong or weak inversion, without affecting circuit operating conditions. In general, NMOS pseudoresistors require less area than PMOS pseudoresistors but generate more noise. The current through the pseudoresistor (Figure 3a) is given by:

(5) $$I_{d}=I_{do}e^{\frac{Vsg}{\eta V_{T}}}\;(1-e^{\frac{-Vsd}{V_{T}}})$$

where

(6) $${\begin{array}{c} \;\\ I \end{array}}_{do}=2µ\eta C_{ox}\frac{W}{L}V_{T}^{2}e^{\frac{{-V}_{To}}{\eta V_{T}}}$$

where µ is the mobility of charge carriers, $C_{ox}$ is oxide capacitance per unit area, η is the sub-threshold slope, $V_{T}$ is the thermal voltage, $\frac{W}{L}$ is the aspect ratio and $V_{To}$ is the threshold voltage.

The resistance of the tunable pseudoresistor (Figure 3b) is given by:

(7) $$R_{pseudo}=\frac{dV_{sd}}{dI_{d}}=\frac{V_{T}}{I_{do}}e^{\frac{-V_{tune}}{\eta V_{T}}}=\frac{L}{4\eta µC_{ox}V_{T}W}\frac{{(e}^{V_{To}-V_{tune}})}{\eta V_{T}}$$

A tuned pseudoresistor in parallel configuration with a drain and a gate terminal tuned with an external voltage is shown in Figure 3c. The structure in Figure 3c is suitable for high-frequency operations and also immune to the effect of parasitic capacitances. The pseudoresistor’s linearity can be improved by using multiple units in parallel, as shown in Figure 3c, making it ideal for use as a feedback resistor in chopper-stabilized amplifiers. The equivalent resistance of this parallel configuration (Figure 3c) is:

(8) $$R_{parallel}=R_{M_{A}}||R_{M_{B}}=\frac{R_{psuedo}}{2}$$

where $R_{psuedo}$ is given in Equation (7).

### 2.3. Core Amplifier

**Figure 4:**
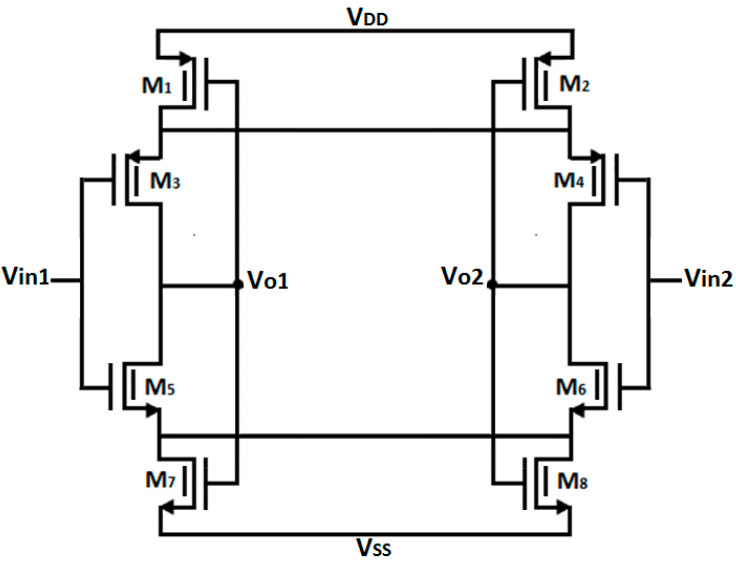
Circuit diagram of inverter-based core amplifier (A).

Inverter-based amplifiers are easily fabricated and have a smaller area footprint, making them suitable for high-frequency applications [18] and comparators [19]. The relationship between high gain and low voltage supply can be managed using a cascade structure, though this reduces output voltage swings. Inverter-based amplifiers offer both high gain and large voltage swings since the overall gain is proportional to the transconductance of both the NMOS and PMOS devices, resulting in a g_m_ [20]. Proper biasing, which ensures both transistors are saturated, is critical for the inverter to function as an amplifier. The gate biasing voltage is largely determined by the output voltage swing and linearity. For an inverter to function as an amplifier, the following condition must be satisfied:

(9) $$V_{\mathrm{DD}}\;>V_{\mathrm{tn}}\;+\;│V_{\mathrm{tp}}│$$

where V_DD_ is the supply voltage, V_tn_ is the threshold voltage of the NMOS, and V_tp_ is the threshold voltage of the PMOS. If the above condition is not satisfied, the transfer curve will become distorted, and the inverter cannot be used as an amplifier. To improve stability, self-biasing the amplifier is beneficial as it makes the amplifier less sensitive to external parameter variations. Common-mode feedback (CMFB) can also be employed to minimize variations due to Process, Voltage, and Temperature (PVT) [21]. The gain and output resistance of the inverter-based amplifier can be expressed as:

(10) $${Av}_{diff}=\;-({g_{m\;5,6}+g}_{\begin{array}{c} m\;3,4 \end{array}})({r_{o\;5,6}//r}_{o\;3,4})$$

(11) $${r_{out}{=r}_{o\;5,6}//r}_{o\;3,4}$$

where *r*_o5,6_ is the output resistance of M_5_ or M_6_, r_o3,4_ is output resistance of M_3_ or M_4_, *g*_m5,6_ is transconductance of M_5_ or M_6_ and *g*_m3,4_ is transconductance of M_3_ or M_4_ in Figure 4.

We designed the current re-use symmetrical amplifier using the inverter topology with FinFETs as the core components, as shown in Figure 4, and denoted as (A). The amplifier circuit consists of a self-biased inverter amplifier with current biasing provided by M_1_, M_2_, M_7_, and M_8_. When a differential voltage is applied to the inverter-based amplifier’s input, either the nFinFET or the pFinFET operates at any given time. The transistors are biased using the g_m_/I_d_ technique, ensuring all transistors operate in moderate inversion for optimal circuit conditions.

The input-referred noise of the inverter-based amplifier includes both flicker noise and thermal noise components. The input-referred noise is given by:

(12) $${\bar{v}}_{ni}^{2}=\frac{8k\cdot T\cdot c}{{g_{m5,6}+g}_{m3,4}}+\frac{2K_{n}}{{W_{3,4.}L}_{3,4.}f\cdot C_{ox.}}+\frac{2K_{p}}{{{W_{5,6.}L}_{5,6.}f\cdot C_{ox}}_{.}}$$

where *c* is the biasing constant, *f* is frequency, *k* is the Boltzmann constant, *C*_ox_ is oxide capacitance, T is the temperature in kelvin, *K*_n_ and *K*_p_ are process-dependent constants, *g*_m5,6_ and *g*_m3,4_ are the transconductances of M_5_ or M_6_ and M_3_ or M_4_, respectively, *W*_3,4_ and *W*_5,6_ are the widths of M_3_ or M_4_ and M_5_ or M_6_, respectively, and *L*_3,4_ and *L*_5,6_ are the lengths of M_3_ or M_4_ and M_5_ or M_6_, respectively.

### 2.4. Feedback Buffer

**Figure 5:**
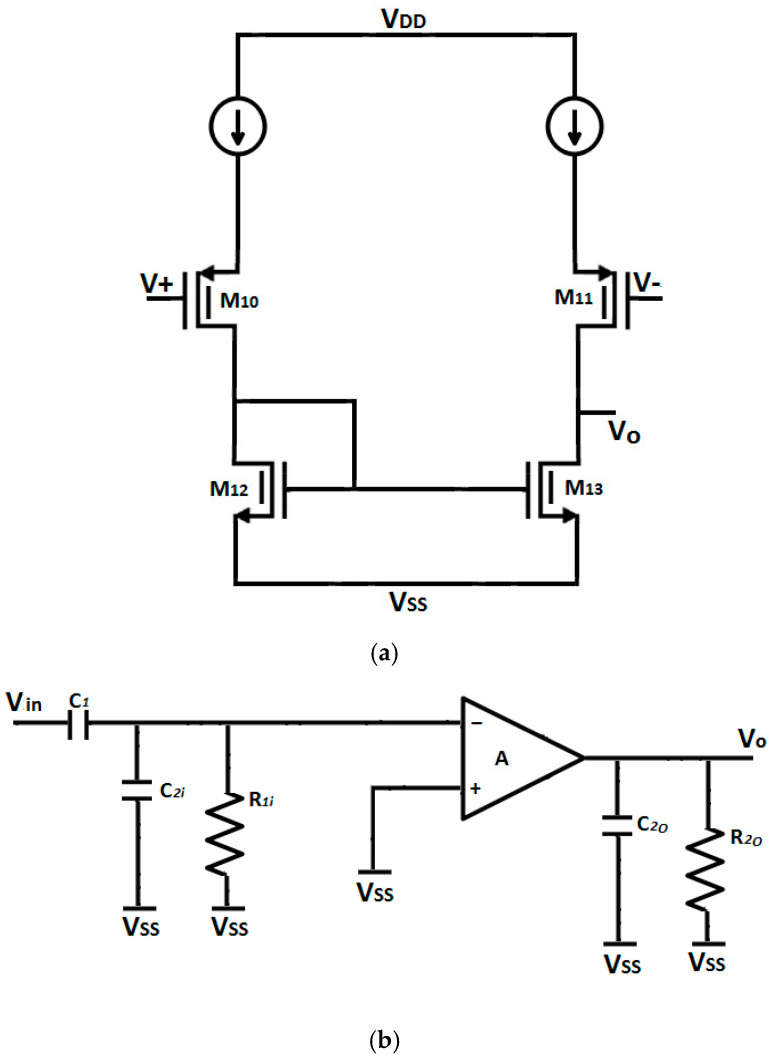
(**a**) Circuit diagram of feedback buffer (β). (**b**) Circuit modification with Miller’s effect on the feedback buffer.

The impedance of the pseudoresistor is sensitive to changes in terminal voltage and PVT variations, leading to poor CMRR performance. The parasitic capacitance of the pseudoresistor impacts both common-mode and differential-mode gain, thereby affecting the CMRR. To mitigate the parasitic capacitance effects, a feedback buffer circuit is placed in parallel with the pseudoresistor, as shown in Figure 5a, which is denoted as (β). The feedback buffer reduces the common-mode gain and enhances the CMRR. As feedback capacitor C_2_ is compensated using Miller’s effect, as shown in Figure 5b, assuming V_ss_ = 0,

(13) $$C_{2i}=C_{2}(1+A)$$

(14) $$C_{2o}=C_{2}(1+\frac{1}{A})$$

where A is the core amplifier gain.

Using the voltage divide rule and assuming *R*_1*i*_ is very large:

(15) $$v^{-}=\frac{C_{1}}{C_{2i}+C_{1}}.v_{in}$$

(16) $$v_{o}=-A\cdot v^{-}$$

Solving both above equations, we get

(17) $$Gain=\frac{v_{o}}{v_{in}}=-\frac{\frac{C_{1}}{C_{2}}}{\frac{C_{1}}{{A\cdot C}_{2}}+\frac{A+1}{A}}$$

The closed-loop gain of the unity feedback buffer is:

(18) $$A_{\beta u}=\frac{\beta }{\beta +1}$$

where β is the feedback buffer gain.

The feedback resistance after Miller’s compensation is given by:

(19) $${\;R}_{1i}=R_{1}(\frac{1}{1-A_{\beta u}})\;{=R}_{1}(1+\beta )$$

(20) $$R_{1}=R_{Ma}||R_{Mb}$$

where $R_{1}$ is the parallel resistance of transistors M_a_ and M_b_, as shown in Figure 3c.

The lower cut-off frequency is

(21) $${ꙍ}_{L}=\frac{1}{R_{1i}.(C_{2i}+C_{1})}$$

(22) $${ꙍ}_{L}=\frac{1}{R_{1}\left(1+\beta \right)\cdot (C_{2}\left(1+A\right)+C_{1})}$$

The lower cut-off frequency, as described by Equation (14), depends on feedback resistance R_1_. The amplification of the feedback resistance also modifies the lower cut-off frequency, which is further enhanced by the bootstrap effect. This allows for coverage of low-frequency components that would otherwise be missed.

### 2.5. Chopper Spike Filter

**Figure 6:**
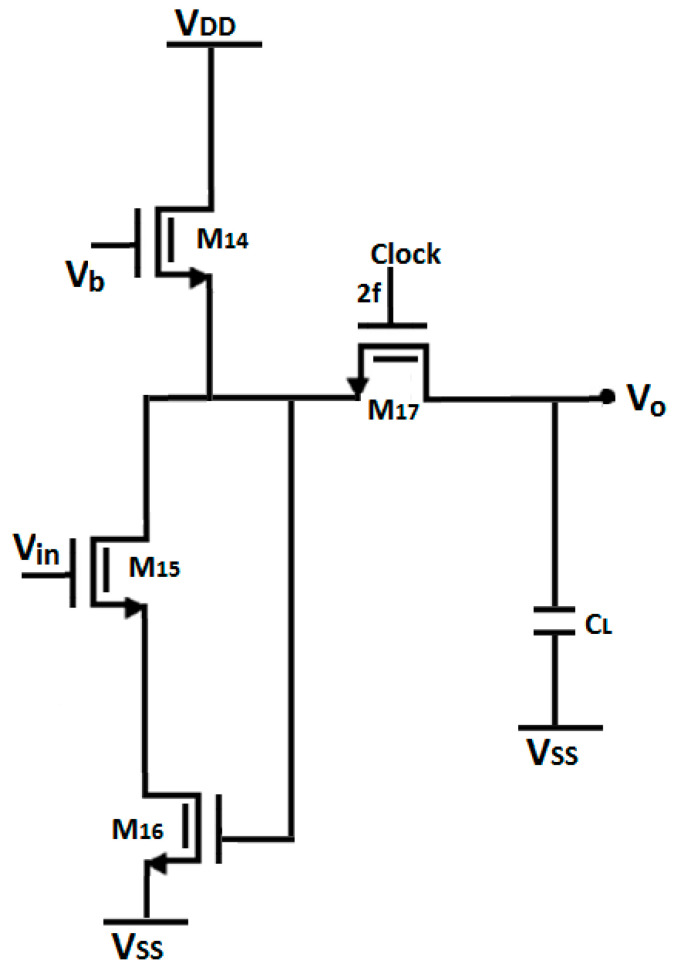
Schematic of chopper spike filter.

A chopper spike filter, shown in Figure 6, is used to reduce spikes generated by switching activities (caused by charge injection). The capacitor absorbs the spikes via the switching activity of M_17_, as shown in Figure 6. The switching frequency applied to M_17_ is double that of f_chop_ (1 kHz), ensuring that M_17_, in combination with C_L_, functions as an R-C filter.

### 2.6. Circuit Design Methodology

The g_m_/I_d_ biasing technique is employed to design the core amplifier. The plot of g_m_/I_d_ vs. I_d_/n_fin_/L is used to set the biasing point, where n_fin_ is the number of fins used in the FinFET. As the minimum n_fin_ value is one, increasing the channel length is necessary to reduce the aspect ratio. In our design, large channel lengths are achieved using the segmentation technique, while keeping the working node within acceptable ranges. The chopper circuit in our amplifier is designed using four FinFETs, driven by non-overlapping clocks at a frequency of 1 kHz, which is 10 times the signal frequency. The proposed amplifier is simulated on HSPICE using 14 nm FinFET technology. The chopping frequency f_chop_ is generally 5–10 times higher than the signal frequency, and in our case, we use *f_chop_* = 1 kHz and a signal frequency of 100 Hz.

## 3. Results

**Figure 7:**
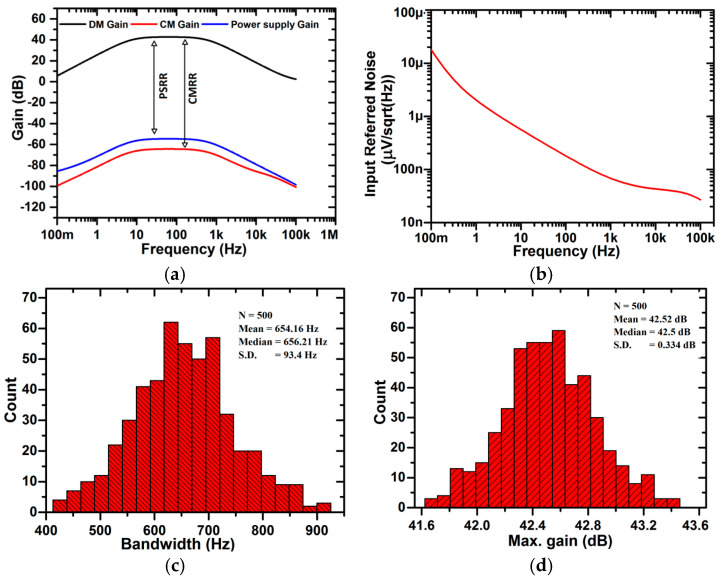

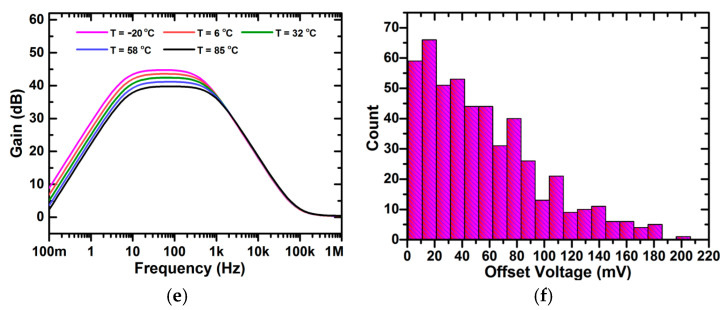
(**a**) Gain vs. frequency curve of chopper-stabilized amplifier. (**b**) Input-referred noise of the chopper-stabilized amplifier (including feedback buffer β), plotted with logarithmic scaling of the y-axis for clearer visualization of the noise spectrum. (**c**) Effect on bandwidth of chopper-stabilized amplifier using Monte-Carlo simulations for 10% statistical variation in supply voltages. (**d**) Effect on mid-band differential mode gain of chopper-stabilized amplifier using Monte-Carlo simulations for 10% statistical variation in supply voltages. (**e**) Gain vs. frequency curve of chopper-stabilized amplifier at different temperatures. (**f**) Monte-Carlo plot of offset voltage for 10% statistical variation in input capacitor (C1).

**Figure 8:**
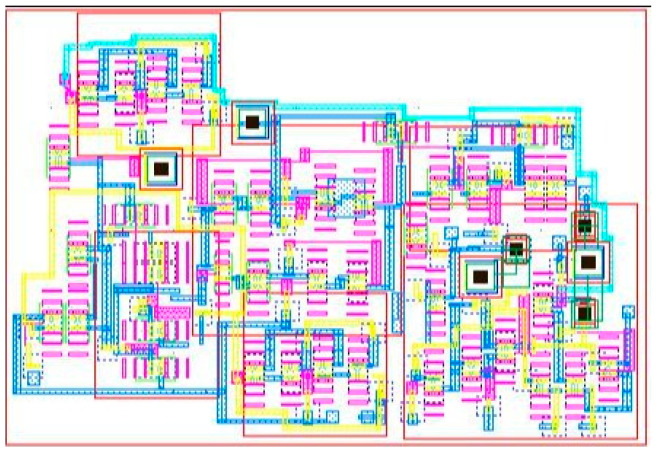
Layout of chopper-stabilized amplifier.

The gain–frequency curve of the proposed chopper-stabilized amplifier is shown in Figure 7a, with a mid-band differential mode gain of 42.6 dB, common mode gain of −64.3 dB, and power supply gain of −54.9 dB. The corresponding CMRR and PSRR values are 106.9 dB and 97.5 dB, respectively. A high CMRR value corresponds to the use of a feedback buffer (β), which decreases the common mode gain and therefore boosts the CMRR, as discussed earlier in Section 2.4. The bandwidth of the amplifier is in the range of 6.96 Hz to 621 Hz. At a V_DD_ of 0.9 V, power dissipation is 921 nW, which is expected as we have used FinFET as the design element. Also, the biasing point of the circuit should be optimal to avoid any extra power requirements while resulting in a good amplification value. The input-referred noise curve for the proposed amplifier is shown in Figure 7b, and the NEF, as calculated by Equation (4), is 6.1. A major contribution to noise comes from the feedback buffer (β), which can be minimized by changing the g_m_ of the input transistors of the feedback buffer (β). Also, a noise contribution comes from the pseudoresistor cell, as all FinFETs in the cell are biased in the sub-threshold region. Figure 7c,d shows the Monte-Carlo simulation with 500 iterations for 10% statistical variation in the values of supply voltages (V_DD_ and V_SS_). In Figure 7c, the mean (σ) and standard deviation (μ) values of the mid-band differential mode gain obtained from the histogram plot are 42.52 dB and 0.334 dB, respectively, which gives a variability (σ/μ) of 0.0078, while in Figure 7d, the mean (σ) and standard deviation (μ) values of bandwidth are obtained as 654.16 Hz and 93.4 Hz, respectively, which gives variability (σ/μ) as 0.143. The results conclude that there is very minimal statistical variation in bandwidth and gain values, showing the robustness of the circuit for parameter variations. Figure 7e shows the variations in gain value in the range of 39.8–44.7 dB while varying the temperature in the range of −20 °C–85 °C, which again proves the robustness of the circuit. Figure 7f presents the Monte-Carlo analysis of offset voltage with 500 runs and 10% variation in the input capacitor (C_1_) value. The offset voltage shows a mean value of 53.24 mV with a standard deviation of 8.3 mV, confirming the robustness of the proposed design against mismatch. The chopper-stabilized amplifier design is further validated through the LVS-verified layout design, as shown in Figure 8. The total area of the layout is 18.2 μm^2^. It should be noted that C_1_ (3800 pF) is realized off-chip with a high-end MLCC capacitor. For a 14 nm FinFET process, the available MIM density can only be 8–10 fF/µm^2^, which implies that on-chip realization of 3800 pF would occupy about 0.4–0.5 mm^2^ of the reserve area—two orders of magnitude more than the amplifier core. Such an implementation would be impractical due to silicon overhead, parasitic loading, and matching issues. Therefore, the given layout area of 18.2 µm^2^ is only associated with the active amplifier core, whereas smaller capacitors like C_2_ are implemented on-chip using MIM structures and are completely part of the given layout.

### 3.1. Effect of the Parallel Cell Configuration of the Tunable Pseudoresistor

**Figure 9:**
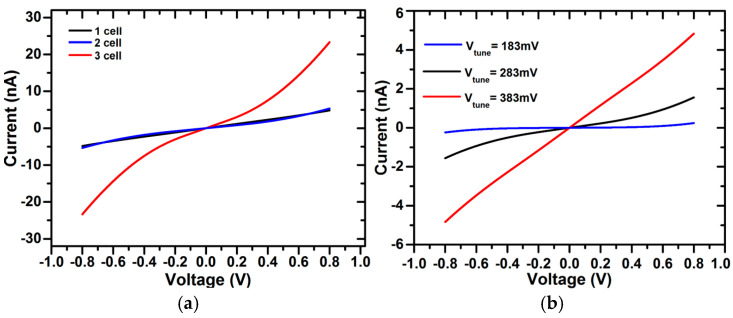
Resistance curve of parallel pseudoresistor cell for (**a**) different numbers of cells and (**b**) different V_tune_ values.

The resistance curve of the parallel cell configuration of tunable pseudoresistors, as discussed in Section 2.2, for different numbers of cells is shown in Figure 9a. The curve shows that the highest linearity is achieved when three cells are used. As the resistance of the pseudoresistor cell depends on the value of V_tune_, as given in Equation (6), the highest linearity of resistance is achieved at V_tune_ = 383 mV, as shown in Figure 9b.

**Figure 10:**
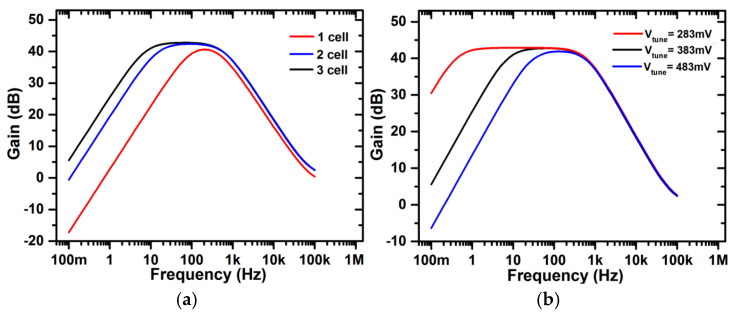
Gain vs. frequency curve of the chopper-stabilized amplifier for (**a**) different numbers of cells and (**b**) for different values of V_tune_.

As the low cut-off frequency of the chopper-stabilized amplifier depends on the value of the feedback pseudoresistor (R_1_) and capacitor, as given by Equation (14), the value of the pseudoresistor (R_1_) in turn depends on the value of V_tune_ and the number of parallel pseudoresistor cells, as discussed previously. The number of parallel pseudoresistor cells and V_tune_ are varied without causing any significant change in gain values, as depicted in Figure 10a,b. The lowest cut-off frequency is achieved when the value of V_tune_ = 283 mV and the number of parallel pseudoresistor cells is 3, as shown in Figure 10. To optimize the circuit design, V_tune_ and the number of cells are selected to provide the highest linearity of the pseudoresistor to avoid any gain change-related distortion while providing a minimum lower cut-off frequency to efficiently process low-frequency biomedical signals. In our design of a chopper-stabilized amplifier shown in Figure 1, we have used three parallel pseudoresistor cells and V_tune_ = 383 mV.

**Figure 11:**
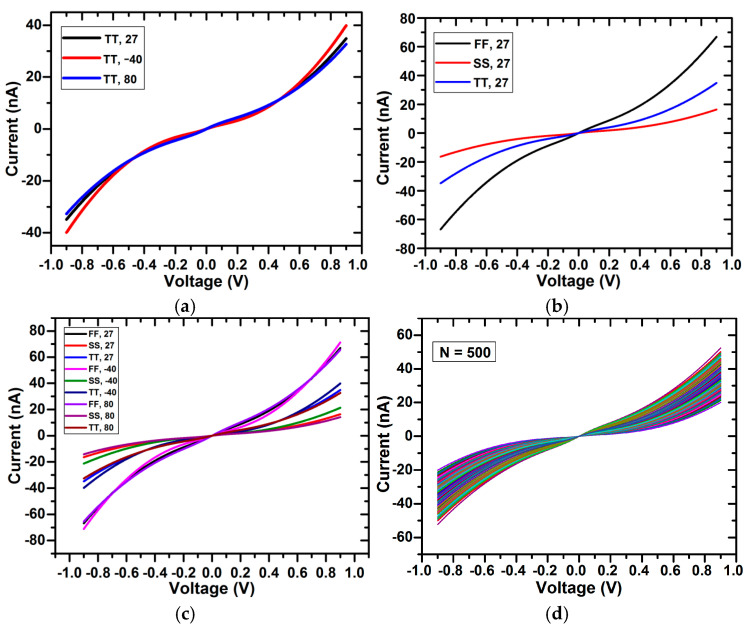
Resistance curve of the parallel pseudoresistor cell for (**a**) different temperatures and the same process corner, (**b**) different process corners and the same temperature, (**c**) different process corners and different temperatures, and (**d**) 500 variations using Monte-Carlo analysis.

**Figure 12:**
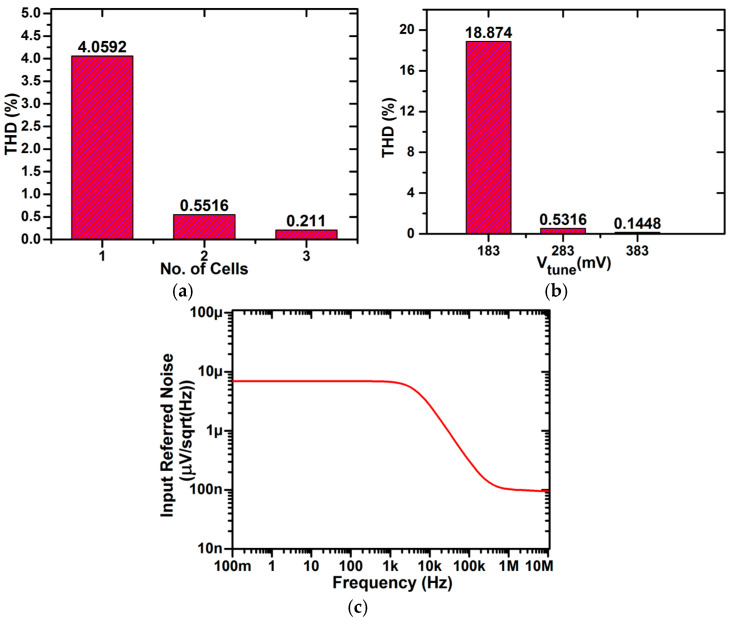
(**a**) THD curve of pseudoresistor cell for different numbers of cells. (**b**) THD curve of pseudoresistor cell for different V_tune_ values. (**c**) Input-referred noise curve of the standalone pseudoresistor cell, explicitly showing its individual contribution to the overall amplifier noise (noise breakdown analysis).

In addition, to investigate the effect of PVT variations on the tunable pseudoresistor cell, the resistance plot for different temperatures, different process corners, and both different process corners and temperature is shown in Figure 11a, Figure 11b, and Figure 11c, respectively. The curves are plotted for TT, FF, and SS process corners. The results show that there is minimal deviation in the resistance curve with process and temperature variations. Also, Monte-Carlo analysis for 500 variations was performed to show the stability performance of the pseudoresistor cell, as shown in Figure 11d. The results show that there are insignificant changes in the resistance values, which again proves the pseudoresistor cell’s immunity against variations. From a theoretical perspective, the improvement in linearity with parallel pseudoresistor cells can be explained using Equation (8), where the equivalent resistance of multiple devices in parallel reduces the nonlinear V_gs_ dependence of a single device. This averaging effect suppresses distortion and leads to better linear behavior. For the linearity assessment of a pseudoresistor cell (Figure 3c), total harmonic distortion (THD) analysis was performed for different numbers of pseudoresistor cells and Vtune values, as shown in Figure 12a and Figure 12b, respectively. It can be clearly observed that, as expected, the THD value decreases with an increase in the number of pseudoresistor cells and also with an increasing Vtune value. The same effect of increasing the number of pseudoresistor cells and Vtune value on linearity can be verified from the current–voltage curve, as shown in Figure 9a,b. In addition, the input-referred noise curve of the standalone pseudoresistor cell is shown in Figure 12c.

### 3.2. Effect of Feedback Buffer

**Figure 13:**
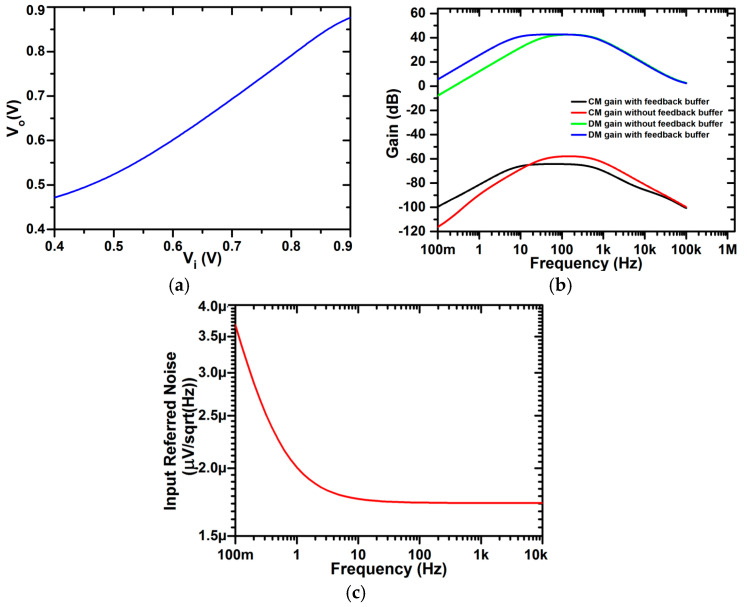
(**a**) Transfer characteristic of the feedback buffer (β), showing that the circuit operates within a sufficiently linear region for the applied input range (0.54 V common-mode bias with 20 mVpp sinusoidal variation). Although not perfectly ideal, this level of linearity is adequate for low-amplitude biomedical signals such as ECGs and EEGs, ensuring distortion-free amplification in practical applications. (**b**) Gain vs. frequency response of the complete chopper-stabilized amplifier (including pseudoresistor, feedback buffer, and chopper spike filter) for common-mode (CM) and differential-mode (DM) signals. The resulting CMRR is 106.9 dB with the feedback buffer (β) and 100.3 dB without it, confirming the buffer’s effectiveness in enhancing performance. (**c**) Input-referred noise curve of the standalone feedback buffer (β), isolating and quantifying its individual contribution to the total amplifier noise (noise breakdown analysis).

As shown in Figure 1, the feedback buffer (β) is used in parallel with pseudoresistor R_1_, where R_1_ is given by Equation (13), to increase the CMRR, as explained in Section 2.4. Figure 5a shows the circuit of the feedback buffer (β), which shows a differential input stage with a current mirror circuit. The transfer characteristic of the buffer circuit proves the linearity of the feedback buffer (β) over a wide range of input voltages, as shown in Figure 13a. Even though the transfer curve is not ideal, the linearity seen in the figure is more than adequate for biomedical purposes because the ECG/EEG signals are within the µV–mV range, and within this input range, the buffer operates reliably without distortion. The input voltage (vin1 and vin2) is indicated in Figure 1, utilizing a DC biasing of 0.54 V with a sinusoidal 20 mV peak-to-peak fluctuation, thus enabling the feedback buffer (β) to quite effectively boost the CMRR value. The results of the common mode and differential gain with and without a feedback buffer are shown in Figure 13b. This plot signifies the CMRR frequency response of the complete chopper-stabilized amplifier (including a pseudoresistor cell, feedback buffer, and chopper spike filter), thereby capturing the full-system behavior. The CMRR measured with and without a feedback buffer is 106.9 dB and 100.3 dB, respectively. It is also evident from Figure 13b that the feedback buffer also reduces the low cut-off frequency, which has advantages in the case of biomedical signals, as they inherently have low frequencies. The low cut-off frequency with and without a feedback buffer is 6.96 Hz and 30.7 Hz, respectively. The feedback buffer is particularly beneficial in neural signal amplification, as it provides a high CMRR and, at the same time, also reduces the low cut-off frequency. In addition to using a feedback buffer (β), the low cut-off frequency can further be reduced by changing the parameters of the pseudoresistor (i.e., V_tune_, number of cells) as discussed previously. Furthermore, to isolate its contribution to the overall amplifier noise shown in Figure 7b, the input-referred noise curve of the standalone feedback buffer (β) is demonstrated in Figure 13c. This allows a clearer evaluation of the individual noise introduced by the buffer.

### 3.3. Effect of the Chopper Spike Filter

**Figure 14:**
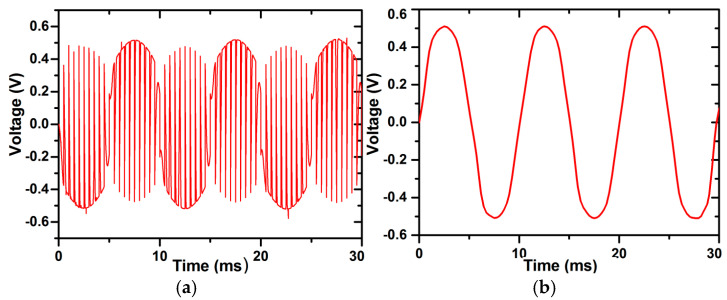
(**a**) Voltage vs. time curve of output signal at chopper spike filter input. (**b**) Voltage vs. time curve of output signal at chopper spike filter output.

The output waveforms at the input of the chopper spike filter and the output of the chopper spike filter are shown in Figure 14a and Figure 14b, respectively. There is a clear trend of a reduction in spikes without distorting the shape of the waveform.

## 4. Machine Learning-Based Application of Biomedical Signals for Arrhythmia Diagnosis

**Figure 15:**
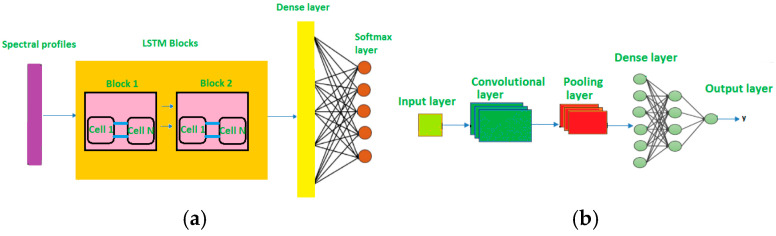
Architecture of (**a**) Long Short-Term Memory (LSTM) and (**b**) convolutional neural networks (CNNs).

Heart disease is one of the leading causes of death worldwide. The electrocardiogram (ECG) is a non-invasive and reliable diagnostic method for heart disease. Early diagnosis reduces the financial burden, especially in low-income countries. Machine learning-based diagnosis of cardiovascular diseases has been widely explored in the literature, and various algorithms have been applied and tested for heart disease diagnosis accuracy. Manual analysis of ECG data is cumbersome, making machine learning-based diagnosis more viable while also aiding in heart disease prediction. Convolutional neural networks (CNNs) have been applied to heart disease diagnosis with 96% accuracy [22]. Additionally, Long Short-Term Memory (LSTM) networks, utilizing the salp swarm algorithm, have achieved an accuracy of 97.11% [23]. The architectures of LSTM and CNN are shown in Figure 15a and Figure 15b, respectively.

To correctly diagnose arrhythmias, we used a CNN+LSTM algorithm. The chopper-stabilized amplifier designed in Section 2.1 was used for conditioning and pre-processing the signals. This combination of a chopper-stabilized amplifier and an arrhythmia classification model in a single system will function as a major Internet of Things (IoT) application for real-time acquisition and processing of biomedical signals and disease classifications. To establish this integration, transient simulations (voltage–time curves) of the proposed chopper-stabilized amplifier are simulated in HSPICE using 14 nm FinFET technology with ECG-like input signals. The amplified outputs, providing sufficient gain and minimal noise/offset, are exported and used as input to the CNN+LSTM pipeline. This ensures the ML classifier is trained and tested on realistically conditioned signals, mimicking a real-world biomedical acquisition system. Although the current classification is offline, it offers a proof of concept for future real-time wearable and IoT healthcare devices. With sufficient gain and offset effects provided by the chopper-stabilized amplifier, the dataset is ready for training and testing. We used 80% of the data for training and 20% for testing. The data was split into five classes according to the AAMI EC57 standard [24]. In this work, both CNN and LSTM algorithms were used to diagnose arrhythmias. The CNN works with 3D data for image and object recognition. While the CNN is powerful, it requires a large dataset for training. The CNN assigns weights and biases to various information sources and can differentiate between them, providing temporal and spatial data auto-extraction. LSTM, a subtype of Recurrent Neural Networks (RNNs), handles time-series data with long-term dependencies and is suitable for real-time signals. It is primarily used for speech recognition and video analysis.

**Figure 16:**
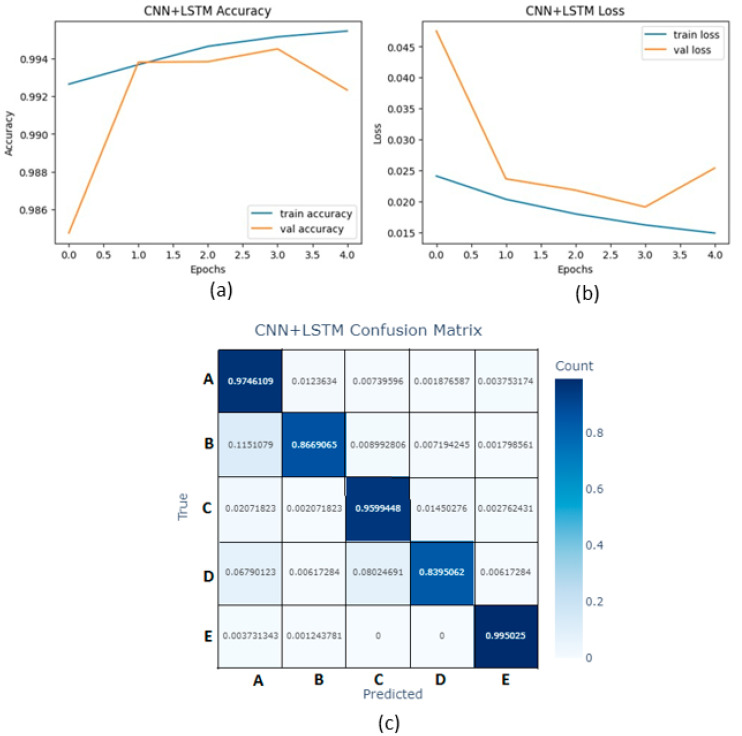
(**a**) Accuracy vs. epochs for the CNN+LSTM algorithm. (**b**) Loss vs. epochs for the CNN+LSTM algorithm. (**c**) Confusion matrix for the CNN+LSTM algorithm.

This work was performed on a computer with a 2.2 GHz processor and 12.6 GB of RAM. After balancing the training dataset, the size was 362,355. A total of 80% of the data was used for training. A plot of accuracy and loss against epochs is shown in Figure 16a and Figure 16b, respectively. The CNN layer in the CNN+LSTM algorithm extracts features from time-series ECG data while the LSTM layer captures the dynamic range of heart rhythms, providing crucial information for diagnosis. For arrhythmia diagnosis using the CNN+LSTM algorithm, an accuracy of 99.12% and a mean square error (MSE) of 0.0273 were achieved. In addition, the confusion matrix of the CNN+LSTM algorithm is shown in Figure 16c.

## 5. Discussion

The proposed chopper-stabilized amplifier, shown in Figure 1, is compared with state-of-the-art works in Table 1. The component dimensions are shown in Table 2. It is important to note that C_1_ (3800 pF) is implemented off-chip with a high-quality MLCC capacitor. On the other hand, smaller capacitors like C_2_ are realized on-chip with MIM structures and are completely included in the reported layout area. The comparison study reveals the following points:

- Higher CMRR: This work presents a better CMRR compared to other works [25,26,27,28,29,30,31], primarily due to the use of a feedback buffer. Unlike [32], where the CM-REP technique was utilized, the proposed approach improves the CMRR by reducing the common-mode gain through parasitic capacitance compensation using the buffer.
- Lower cut-off frequency: In this work, a reduced low cut-off frequency is achieved by applying Miller’s effect simultaneously on both the feedback buffer and capacitance, while in [31], Miller’s effect is applied only on the feedback capacitor.
- Technology advancement: The proposed amplifier is designed using novel FinFET technology (14 nm), whereas prior works [25,26,28,29,30] relied on conventional CMOS technology, leading to a higher power consumption.
- Tunable pseudoresistor: This work introduces a tunable pseudoresistor with multiple cells, which allows for precise control over linearity and cut-off frequency. In contrast, earlier works [26,27,28,29,30] used non-tunable pseudoresistors or large resistors, limiting flexibility.
- Biasing technique: The g_m_/I_d_ biasing technique is used in this work, with the circuit biased in the moderate inversion region, resulting in an optimal trade-off between power, gain, and noise.
- Interdisciplinary application: In addition, this study presents a machine learning-based proof of concept for arrhythmia classification, which demonstrates the amplifier’s practical usefulness in real-time biomedical IoT applications.
- Robustness to mismatch: The Monte-Carlo offset analysis (Figure 7f) exhibits a mean value of 53.24 mV and a standard deviation of 8.3 mV, producing a variability ratio (σ/μ) of 0.155. This is lower than the typically reported values in CMOS-based bio-amplifiers [25,26,28], confirming that the proposed FinFET-based design provides better robustness against process variation and mismatch.

## 6. Conclusions

In this paper, a chopper-stabilized capacitively coupled amplifier is designed using a novel tunable pseudoresistor cell and a feedback buffer circuit. The tunability of the pseudoresistor is shown by varying the tuning voltage (V_tune_) and the number of pseudoresistor cells. This tunability can control various amplifier characteristics, providing fine control over the circuit. The feedback buffer is employed in a parallel configuration to reduce common-mode gain, resulting in a high CMRR. It has been shown that the feedback buffer also reduces the low cut-off frequency. A chopper spike filter is used at the amplifier’s output terminal to eliminate spikes generated during the switching process. Moreover, Monte-Carlo analysis confirms that the proposed FinFET-based design exhibits lower variability than conventional CMOS amplifiers, ensuring enhanced robustness for real-world biomedical applications. Future work could focus on enhancing the CMRR at ultra-low frequencies and minimizing the noise introduced by the feedback buffer. Overall, the proposed architecture is different from previous architectures in the respect that it employs a tunable pseudoresistor to achieve improved linearity, a feedback buffer (β) to achieve simultaneous CMRR enhancement and cut-off frequency reduction, and an ultra-low-power FinFET implementation, thereby making it very flexible for future wearable and IoT-based biomedical applications.

## Figures and Tables

**Table 1:** Comparison of the proposed chopper-stabilized amplifier with state-of-the-art works.

Reference	Technology	Channel Length (nm)	Amplifier Gain (dB)	Bandwidth (kHz)	Supply Voltage (V)	Power (µW)	CMRR (dB)	NEF
[25]	CMOS	180	47.6	0.5	1.5	0.85	105.6	2.91
[26]	CMOS	180	60	--	1	0.33	60	11
[27]	FinFET	30	81.37	--	1	388	127.6	9.63
[28]	CMOS	180	35	0.2	±0.6	0.64	>100	9.11
[29]	CMOS	130	27–39	7.5	0.8	1.6	67	1.62
[30]	CMOS	40	89	--	0.5	0.72	101	1.03
[31]	CMOS	350	40	20	3.3	6.7	90	1.8
[32]	CMOS	180	46–64	--	1.8	--	>130	2.34
[This Work]	FinFET	14	42.6	0.61	0.9	0.92	106.9	6.1

**Table 2:** Dimensions of the components used in the circuit.

Component	Dimensions
M1, M2, M3, M4	1/450 nm (nfin/L)
M5, M6, M7, M8	1/1800 nm (nfin/L)
M10, M11	1/10 um (nfin/L)
M12, M13	1/68.8 um (nfin/L)
*C*_1_	3800 pF
*C*_2_	30 pF

## Data Availability

The detailed design methodology and the used design parameters are presented in the article. No additional data sharing is applicable to this article.
